# A type IVB pilin influences twitching motility and *in vitro* adhesion to epithelial cells in *Burkholderia pseudomallei*

**DOI:** 10.1099/mic.0.001150

**Published:** 2022-03-16

**Authors:** Udoka Okaro, Sherry Mou, Geraldin Lenkoue, Janice A. Williams, Ari Bonagofski, Peter Larson, Raina Kumar, David DeShazer

**Affiliations:** ^1^​ Bacteriology Division, United States Army Medical Research Institute of Infectious Diseases, Frederick, Maryland, USA; ^2^​ Pathology Division, United States Army Medical Research Institute of Infectious Diseases, Frederick, Maryland, USA; ^3^​ Molecular Biology Division, United States Army Medical Research Institute of Infectious Diseases, Frederick, Maryland, USA

**Keywords:** melioidosis, type IV pili, biofilm formation

## Abstract

Type IV pili are involved in adhesion, twitching motility, aggregation, biofilm formation and virulence in a variety of Gram-negative bacteria. *Burkholderia pseudomallei,* the causative agent of melioidosis and a Tier 1 biological select agent, is a Gram-negative bacterium with eight type IV pili-associated loci (TFP1 to TFP8). Most have not been fully characterized. In this study, we investigated *BPSS2185*, an uncharacterized TFP8 gene that encodes a type IVB pilus protein subunit. Using genetic deletion and complementation analysis in *B. pseudomallei* JW270, we demonstrate that *BPSS2185* plays an important role in twitching motility and adhesion to A549 human alveolar epithelial cells. Compared to JW270, the JW270 *ΔBPSS2185* mutant failed to display twitching motility and did not adhere to the epithelial cells. These phenotypes were partially reversed by the complementation of *BPSS2185* in the mutant strain. The study also shows that *BPSS2185* is expressed only during the onset of mature biofilm formation and at the dispersal of a biofilm, suggesting that the motility characteristic is required to form a biofilm. Our study is the first to suggest that the *BPSS2185* gene in TFP8 contributes to twitching motility, adhesion and biofilm formation, indicating that the gene may contribute to *B. pseudomallei* virulence.

## Data Summary

*Burkholderia pseudomallei* is the causative agent of melioidosis and is a Tier 1 biological select agent. A previous study found that the TFP8 genes were important for *B. pseudomallei* virulence in a murine model of infection, but the function of the encoded pilus was not further evaluated. Our research shows that TFP8 plays an important role in attachment, twitching motility and biofilm formation, all of which can probably affect virulence.

## Introduction

*Burkholderia pseudomallei* is an opportunistic, motile, Gram-negative, environmental saprophyte that carries a wide range of virulence factors and broad antimicrobial drug resistance features [1]. In humans it is the causative agent of melioidosis and is a Tier 1 biological select agent because it poses a severe threat to both human and animal health. The clinical presentation of the disease, which is endemic in Southeast Asia and Australia, can range from an acute febrile illness to a disseminated septicaemia. Long incubation periods before clinical symptoms are possible and recurrence of infection is common despite adequate antimicrobial therapy [1]. The disease is probably under-recognized and under-reported. Based on modelling, the disease may be responsible for up to 165000 cases per year, with 89000 deaths [2]. Recent years have seen an upsurge in *B. pseudomallei* research because of its potential use as a biological warfare agent and increasing awareness of the disease and its health burden [1–3]. Understanding the mechanisms that contribute to its virulence, allow it to evade the immune system and develop antimicrobial resistance is essential for identifying potential medical countermeasures. Adhesion, twitching motility and biofilm production mediated by type VI pili, which are present in *B. pseudomallei* and other Gram-negative bacteria, play an important role in their ability to cause disease [4, 5].

Most bacteria have multiple types of pili, which are categorized into different classes based on assembly processes or pathways [6]. Type IV pili (TFP) are long and thin surface filaments that function by repeated extension and retraction [7, 8]. They are often involved in diverse processes such as attachment, twitching motility, DNA uptake, biofilm formation and virulence. There are two distinct subclasses of TFP, termed type IVA and type IVB, that are differentiated by the length of pilin signal peptides and other conserved motifs [8]. *B. pseudomallei* encodes eight type IV pilus-associated loci, TFP1 to TFP8, and both type IVA and IVB pili genes are represented [9]. The TFP1 locus contains a type IVA gene that is involved in adherence to human epithelial cell lines, and virulence in nematode worms and mice. Strain differences exist with regard to the role of TFP1 in adherence, microcolony formation and expression of the pilus subunit gene *pilA* [10]. Nandi *et al*. found that the TFP2, TFP4 and TFP7 loci are positively selected for and may confer an advantage in environmental survival and host adaptation [11]. When the *B. pseudomallei* TFP4 locus was deleted, the mutant exhibited significantly reduced virulence in the BALB/c mouse intranasal infection assay. The type IVB pilus subunit, PilV, encoded by TFP7 was immunogenic as a vaccine candidate in mice, but was unable to induce protection against *B. pseudomallei* challenge [12]. Finally, the TFP8 locus was identified as a recombination hotspot in *B. pseudomallei* and a deletion of the TFP8 genes resulted in a strain that exhibited reduced virulence in a BALB/c mouse intranasal infection assay [13]. The role of TFP8 in other phenotypes, such as adherence, twitching motility and biofilm formation, was not evaluated. These factors probably play a role in colonization of hosts and disease causation.

The adhesion process is critical for a bacterium’s capacity to colonize, infiltrate and cause disease [14]. Gram-negative bacteria can adhere to surfaces using trimeric auto transporters [15], outer membrane proteins [5], filamentous haemagglutinins [16], polysaccharide adhesins [17] and pilus proteins [4]. The pili adhesins are polymeric fibres that are used for biotic and abiotic surface attachment, twitching motility, DNA transfer, biofilm development and transitioning from planktonic to sessile growth [18]. Bacterial adhesion and aggregation are the first stages of biofilm formation, followed by maturation and dispersion to form a new biofilm [19]. Adhesion, aggregation and biofilm formation can be mediated by flagella [20], fimbriae [21], type I pili produced by the CUP pathway [21] or type IV pili [20], or by the presence of various chemical compositions such as extracellular DNA (eDNA) [22, 23] or polysaccharide [24] on the surface of the bacteria. Biofilm production in *B. pseudomallei *, *Staphylococcus aureus* and *Yersinia pseudotuberculosis* has been linked to the higher mortality in both *Caenorhabditis elegans* and mouse models [25–27], and has been implicated in immune responses [26]. After a matured biofilm is formed, bacteria are released and move to a different location to form a new biofilm [28–30]. Biofilm formation in a *B. pseudomallei* infected host often results in persistent bacteraemia as cells are continually disseminated *in vivo* and complete eradication becomes difficult [26].

The role of the TFP8 locus in mediating adherence, twitching motility and biofilm formation has not been described in *B. pseudomallei *. The objective of our study was to use genetic deletion and complementation analysis in *B. pseudomallei* JW270 to characterize the contribution of TFP8 to virulence.

## Methods

### Bacterial strains

Table 1 identifies the strains of bacteria, constructs, plasmids and primers used in this study and their relevant characteristics. *B. pseudomallei* JW270 was selected because it is an attenuated strain that is exempt from the Select Agent regulations. *Escherichia coli* strains used for mutant constructs were grown in Lennox LB broth (Sigma-Aldrich) at 37 °C with shaking overnight. *E. coli* strains containing pBHR2 and pBHR2-*BPSS2185* were grown in LB broth supplemented with 50 µg kanamycin ml^−1^ (Sigma-Aldrich).

**Table 1.** Bacterial strains, plasmids and primers used for this study

Strain/plasmid/primer (5′−3′)	Relevant characteristics	Source or reference
* E. coli* INV110	For growth and purification of plasmid DNA digested with *dam* or *dcm* methylation-sensitive restriction enzymes	Invitrogen
* E. coli* TOP10	General cloning and blue/white screening	Life Technologies
* E. coli* S17-1	Mobilizing strain, transfer genes of RP4 integrated in chromosome; Sm^r^	[51]
pMo130	Km^r^, *Burkholderia* gene replacement vector	[34]
pBHR2	Broad-host-range plasmid, Km^r^	[35]
pBHR2-*BPSS2185*	pBHR2 containing a full-length copy of *BPSS2185*	This study
* B. pseudomallei* JW270	Strain excluded from the CDC select agent list as a result of a deletion of the *wcb* locus encoding the 6-deoxyheptan capsular polysaccharide; *∆*(*amrR-oprA*) *rpsL* (Sm^r^)	[52]
* B. pseudomallei* JW270 *ΔBPSS2185*	JW270 derivative with an in-frame 138 bp deletion of *BPSS2185*	This study
*BPSS2185* F1	*TTAT ***CATATG*** GAATACCGGGGTAA	This study
*BPSS2185* R1	GAGGCTGGACATGACT	This study
*BPSS2185* F2	AGCCTCACGATCGGCAACGA	This study
*BPSS2185* R2	*TGTA ***GGATCC*** GAGCGGAAAGCAT	This study
C-*BPSS2185* F	*TTA ***CATATG*** ACGGCAAGGGTTTTA	This study
C-*BPSS2185* R	*TATA ***GGATCC*** ACGCACCCTTACA	This study

Km^r^, kanamycin resistant; Sm^r^, streptomycin resistant.

*Restriction enzyme sites are bolded, italicized and underlined. CATATG – *Nde*I, GGATCC – *Bam*HI.

For the adhesion, motility and biofilm experiments, *B. pseudomallei* JW270 and *B. pseudomallei* JW270 *ΔBPSS2185* were cultured on LB agar, and JW270 *∆BPSS2185* (pBHR2) and JW270 *ΔBPSS2185* (pBHR2-*BPSS2185*) were cultured on LB agar with 50 µg kanamycin ml^−1^. Overnight colonies were transferred into 1 ml of LB and LB with 50 µg kanamycin ml^−1^. Unless otherwise stated, these *B. pseudomallei* strains were grown overnight in a 37 °C incubator with shaking at 250 r.p.m. then transferred to a 24-well plate containing 2 ml of LB broth and grown as a biofilm without agitation for 1–3 days. The strains were subsequently serially diluted, plated on LB agar to determine the bacterial concentration and used for experiments.

### RNA-sequencing (RNA-Seq) and transcriptomic analysis

Bacterial aggregation, biofilm formation and extracellular DNA production have been linked to the presence of calcium and divalent cations [31, 32]. Environmental calcium was shown to regulate production of a Type IV Tad pilus responsible for biofilm formation, aggregation and colonization by *Vibrio vulnificus* [31]. *B. pseudomallei* JW270 was inoculated in six T175 cell culture flasks, two for each experiment containing either 50 ml LB broth or LB broth supplemented with 1.26 mM CaCl_2_. Cultures were grown statically for 48 h at 37 °C. Spent medium was removed and the biofilm was washed twice with 20 ml of sterile 1× PBS (Sigma-Aldrich). RNAs from six independent samples of bacteria grown in LB or LB with CaCl_2_ were extracted using the Direct-zol RNA MiniPrep kit (R2052; Zymo Research), according to the manufacturer’s protocol. The six extracted RNA samples were prepared using the TruSeq Stranded Total RNA Library Prep Kit (Illumina). RNA samples were diluted to 100 ng µl^−1^ in 10 µl of ultrapure water and prepared as directed by the manufacturer. rRNA was depleted using the RRM G Mix (Illumina). Adapter ligation libraries were quantified using a KAPA Biosystems Library Quantification Kit (Roche Sequencing Systems) with the KAPA SYBR FAST qPCR Master Mix and assessed on a Tapestation 2200 (Agilent) for library size and integrity. All six libraries were pooled at equimolar concentrations yielding a 2 nM pool that was spiked with a 10 % Illumina PhiX loading control. The pooled library was diluted to 14 pM and sequenced using a Paired-End HiSeq Rapid SBS Kit (V2-500 cycles) on a HiSeq 2500 System (Illumina) following the manufacturer’s methods. The sequencing run yielded approximately 300 million reads with a Q30 >83 %. The RNA-Seq data were deposited in Figshare (accession number: https://figshare.com/articles/dataset/Burkholderia_pseudomaelli/17704376).

### Statistical and bioinformatics analysis for RNA-Seq data

The raw reads with low-quality ends that had a Phred score of <Q20 were removed from the analysis. Ligated adaptor sequences were trimmed using Trimmomatic [33] needed to check with following filters of a minimum length of 80 bp. Quality reads were further aligned to the *B. pseudomallei* 1026b reference genome (NC_017831.1 and NC_017832.1) using Spliced Transcripts Alignment to a Reference (STAR) software (GitHub). The total number of raw reads sequenced per sample averaged 47 million and quality filtered reads averaged 14 million. An mRNA read count matrix for each sample was created by identifying the best hits to the genome using featureCounts R (Bioconductor) against the reference gene feature annotation table from the UCSC database. On average, 25 % of the sequence reads were mapped to the *Burkholderia* genome. The gene-specific library size depths were in the range 3–6 million. Read counts were further filtered with a read count cutoff of <0.5 counts per million across all sample populations to filter out the zero read count mRNAs. Hits for the top genes were confirmed using RT-qPCR. Genes with high expression profiles were selected for further studies.

For the detection of differentially expressed mRNA between bacteria grown in LB media – samples M1, M2 and M3 – to bacteria grown in LB media with CaCl_2_ – samples C1, C2 and C3 – the read counts were normalized using the Trimmed Mean of M values (TMM) followed by differential expression analysis using the edgeR software package (Bioconductor). Statistically significant differentially expressed mRNAs were identified using empirical Bayes quasi-likelihood F-tests, with the contrast function (samples M1, M2 and M3 compared with samples C1, C2 and C3), after fitting the quasi-likelihood negative binomial generalized log-linear model on counts. Statistically significant differences in mRNA had *P*-values of <0.05 and false discovery rates (FDRs)<0.05. Differentially expressed genes with *P*-values of <0.05 were selected for functional gene ontology enrichment analysis using the LIMMA R package (Bioconductor). Based on the results of this analysis, the *B. pseudomallei* JW270 gene encoding *BPSS2185* was selected for use in the following experiments.

### Construction of JW270 *ΔBPSS2185* and JW270 *ΔBPSS2185* (pBHR2-*BPSS2185*)

To delete the *BPSS2185* gene, genomic DNA was extracted from the overnight growth of JW270 cells using the GenElute Bacterial Genomic DNA kit (Sigma-Aldrich), according to the manufacturer’s protocol. PCR primer pairs 2185 F1 and R1 and 2185 F2 and R2 were used to amplify 450 bp upstream and downstream of the *BPSS2185* gene. The PCR product was cleaned and purified using the QIAquick PCR Purification Kit (Qiagen) according to the manufacturer’s protocol. Primer pair *BPSS2815* F1 and *BPSS2185* R2 were used for overlap PCR. The final product of about 900 bp was purified, digested with *Nde*I and *Bam*HI, and gel-purified using 0.7 % agarose gel. The amplicon was ligated to a pMo130 plasmid [34] previously digested with *Nde*I and *Bam*HI. The plasmid was transformed in *E. coli* TOP10 and positive colonies were selected in the presence of 100 µg kanamycin ml^−1^ and verified using PCR and plasmid sequencing. A positive colony was grown overnight, and the plasmid was purified, electroporated into *E. coli* S17-1 and incorporated into *B. pseudomallei* JW270 through conjugation. Transconjugants were selected by plating on LB agar supplemented with 50 µg kanamycin ml^−1^ and 25 µg polymyxin B ml^−1^. Colonies were counterselected on agar containing 10 % sucrose for a second crossover event, resulting in the full-length gene being replaced with the truncated version producing *B. pseudomallei* JW270 *ΔBPSS2185*. The deletion mutant was confirmed by PCR.

To complement the deleted *BPSS2185* gene, primer pairs C-*BPSS2185* F and R were used to amplify the 218 nt product of *BPSS2185*. The amplicon was purified, digested with *Bam*HI and *Nde*l enzymes, and gel-purified with the PureLink Quick Gel Extraction Kit (Invitrogen). The purified amplicon was ligated into the pBHR2 plasmid using the *Bam*HI and *Nde*l sites. The plasmid pBHR2 is a medium-copy broad-host-range plasmid that contains a multiple cloning site downstream of a *cat* promoter that drives the constitutive expression of cloned genes [35]. The plasmid was transformed into chemically competent *E. coli* TOP10 cells (Life Technologies) and the resulting positive colonies were selected using 50 µg kanamycin ml^−1^. Colonies were grown in 5 ml of LB media supplemented with 50 µg kanamycin ml^−1^, and plasmid extraction was done using the PureYield Plasmid Miniprep System (Promega). Sequences were verified by plasmid sequencing and PCR. The plasmid was electroporated into *B. pseudomallei* JW270 *ΔBPSS2185* using a 0.1 cuvette (620; BTX) and pulsed for 4.5 ms and 200 Ω with a constant capacitance of 25 µF. Electroporated cells were incubated in a six-well plate (Thermo Fisher Scientific) containing 1 ml of LB medium for 6 h. Cells were plated on LB agar supplemented with 50 µg kanamycin ml^−1^ to produce *B. pseudomallei* JW270 *ΔBPSS2185* (pBHR2-*BPSS2185*).

### Adhesion to epithelial cells

To determine if BPSS2185 has an adherence function, we investigated the rate of adhesion to a biotic surface – A549 epithelial cells (American Type Culture Collection) – and an abiotic surface using a 96-well polystyrene plate (Corning). Freshly cultured A549 epithelial cells in high glucose Dulbecco’s modified Eagle Medium (Hyclone Laboratories) supplemented with 10 % heat-inactivated bovine serum (without antibiotics) were grown to 90 % confluence at 37 °C and 5 % CO_2_. Cells were washed three times with warm Dulbecco’s PBS (DBPS) (Gibco Laboratories) and incubated with 1 : 10 µl of bacteria overnight growth of *B. pseudomallei* JW270, *B. pseudomallei* JW270 *ΔBPSS2185* and *B. pseudomallei* JW270 *ΔBPSS2185* (pBHR2-*BPSS2185*) diluted in LB broth. Diluted bacterial samples were plated to determine the initial c.f.u. of inoculum. A549 cells grown without bacteria inoculum were used as a control. Bacterial cells and A549 were incubated for 3 h at 37 °C and 5 % CO_2_. Spent medium was removed and cells were washed three times with 1 ml of warm DBPS (Gibco Laboratories). Cells were detached and lysed using 100 µl of 1 % Triton X-100 (Sigma-Aldrich), incubated for 10 min at room temperature, and the reaction was then terminated by adding 900 µl of LB medium. One hundred microlitres of serially diluted samples was plated and incubated overnight at 37 °C. Colonies were counted and the percentage adhered was calculated by dividing the number of c.f.u. of adhered bacteria by the number of c.f.u. of the inoculum.

### Twitching motility assay

To examine the effect of BPSS2185 on *B. pseudomallei* twitching motility, JW270, JW270 *ΔBPSS2185*, JW270 *ΔBPSS2185* (pBHR2) and JW270 *ΔBPSS2185* (pBHR2-*BPSS2185*) were inoculated overnight in 1 ml LB medium with corresponding antibiotics. One hundred microlitres of the overnight inoculum was diluted to an OD_600_ of 0.45 and the bacteria were inoculated with a toothpick onto LB agar plates containing 1 % agar. The plates were incubated at 37 °C for 72 h, and the diameter of the white halo corresponding to the spread of bacteria from the point of inoculation was recorded every 24 h.

### Scanning electron microscopy

Samples were grown either on a coverslip or nitrocellulose membrane (Thermo Fisher Scientific). Each coverslip and nitrocellulose membrane was processed at either 3, 24 or 72 h. The samples were washed with 0.1 M sodium cacodylate buffer (EMSciences) and then fixed with 2.5 % glutaraldehyde and 2 % paraformaldehyde in 0.1 M sodium cacodylate buffer for 1 h prior to submission to the electron microscopy facility. Samples were buffer washed, postfixed with 1 % osmium tetroxide and dehydrated through an ethanol series [30, 50, 75, 85, 95%, three changes of 100%, and a single 1 : 1 with 100 % ethanol and hexamethyldisilazane (HMDS)] for 10 min each. Samples were left to dry in 100 % HMDS for 24 h under a chemical fume hood and imaged with a Sigma VPFE microscope (Carl Zeiss Microscopy).

### Biofilm assay

Biofilms were monitored using crystal violet (CV) in a 96-well polystyrene plate (Corning) as previously described [36]. Approximately 10^5^ bacterial cells of JW270 and the two JW270 constructs were added to 2 ml of LB broth or LB broth supplemented with 50 µg kanamycin ml^−1^. Triplicates of 200 µl per well were cultured into three 96-well plates at 37 °C for 1–3 days. The absorbance (OD_600nm_) of the plates was read daily to determine growth at OD_600nm_. After 24 h, 0.25 µg of DNase was added. At the end of the experiment, the spent medium was discarded. The wells were gently washed twice with 200 µl of 1× PBS. One hundred microlitres of 0.1 % CV solution (Sigma-Aldrich) was added for 15 min and air-dried overnight in a biosafety cabinet. Biofilm was extracted using 200 µl of 30 % acetic acid and quantified at an optical density of 550 nm (OD_550nm_) on a SpectraMax M5 (Molecular Devices).

### eDNA export

To quantify the amount of eDNA exported, approximately 10^5^ bacteria were added to 3 ml of LB media (MBLE-7030, Molecular Biologicals International) containing 1 µl propidium iodide (PI; Invitrogen). Propidium, an impermeable DNA-binding dye that binds eDNA or impaired cells and emits a red fluorescence, was added to monitor eDNA export [37]. Two hundred microlitres of the mixture was dispensed in six replicates and cultured statically on a 96-well plate (Corning) for 3 days in a SpectraMax M5 sample chamber at 37 °C. Two hundred microlitres of LB plus propidium iodide was used as a blank. eDNA export was measured every 30 min by excitation at 535 nm and measuring the fluorescence at 620 nm. Experiments were repeated three times independently.

### Live/dead imaging

Confocal laser scanning microscopy imaging was used to assess the live/dead ratio of *B. pseudomallei* strains, and the cells were inoculated as described in the biofilm assay protocol. Bacteria were inoculated in 96-well plates, gently washed and exposed to molecular dyes. The Filmtracer LIVE/DEAD Biofilm Viability Kit Film (Invitrogen L10316) was used to stain bacteria in the wells according to the manufacturer’s protocol. The bacterial cells were washed with sterile water and imaged immediately using a Nikon Eclipse 90i microscope.

### *BPSS2185* RNA expression

To examine the expression of *BPSS2185* in a biofilm, *B. pseudomallei* was cultured in LB media at 37 °C for 3 days on a six-well polystyrene plate (Corning). The supernatant was carefully aspirated to prevent biofilm disruption. The biofilm was gently washed twice with PBS and the RNA was extracted by directly adding Trizol reagent (Invitrogen) to the plate. Ten micrograms of the resulting RNA was treated with Turbo DNase (Invitrogen), and 1 µg was reverse transcribed to cDNA using the iScript cDNA synthesis kit (Bio-Rad Laboratories). qRT‐PCR was performed in a total volume of 25µl, which consisted of 12.5 µl of the 2× Maxima SYBR Green/Fluorescein qPCR kit (Thermo Fisher Scientific), 300 nM of the forward and reverse BPSS2185 primers (C-BPSS2185 f and rev), and 2 µl cDNA. The 50S ribosomal protein L4 (RplD) (P60723; UniProt Consortium) was used as the reference gene for normalization. The reaction conditions were as follows: a single cycle of 95 °C for 3 min, 40 cycles of 95 °C for 10 s and 60 °C for 30 s, followed by 95 °C for 45 s and 55 °C for 1 min. Melt curve analysis was used to confirm that primer dimers were not generated. The comparative Ct method was used to analyse the data.

### Statistical analysis for independent experiments

All experiments were independently repeated three times. Unless indicated, graphs represent the mean of three independent experiments. Each biofilm assay and export experiment was conducted in triplicate and mean values were compared between groups using Student’s *t*-test. Error bars represent the standard error of the mean. Graphpad Prism 9 (GraphPad Software) was used for statistical analysis. Differences were statistically significant at *P*<0.05.

## Results

### Five genes encoding outer membrane proteins were upregulated in the presence of CaCl_2_

**Fig. 1.**
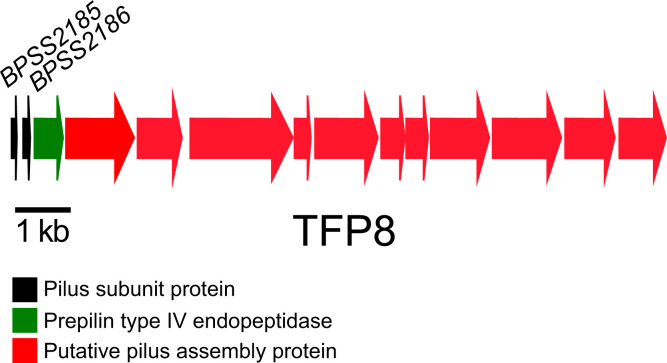
Genetic map of *B. pseudomallei* TFP8, locus tags *BPSS2185–BPSS2198* (K96243) and *BP1026B_II2352-BP1026B_II2365* (1026b). The location and direction of transcription of genes are represented by arrows. The putative functions of the proteins encoded by genes are shown in the colour-coded key below. A 1 kb scale is shown below the genetic locus.

To identify *B. pseudomallei* genes involved in adhesion, aggregation and biofilm formation, JW270 cells grown in LB +1.26 mM CaCl_2_ were analysed for transcriptomic analysis using RNA-Seq and compared to JW270 cells grown in LB. The transcriptomic data identified five genes with at least a 1.5 log change that encoded outer membrane proteins (Table 2). *BPSS2185* and *BPSS2186*, two TFP8 genes that encode type IVB subunit proteins, were highly upregulated (Fig. 1). The other three upregulated genes included two that code for fimbriae-related chaperone proteins, *BPSS0092* and *BPSL1627*, and a gene that encodes a transport-related membrane protein (*BPSS0019*). We focused on type IVB genes, *BPSS2185* and *BPSS2186*, but we were only able to construct an in-frame deletion mutation of *BPSS2185*. The full-length *BPSS2185* gene was cloned into the broad-host-range plasmid pBHR2 and used for complementation studies. JW270, JW270 *ΔBPSS2185*, JW270 *ΔBPSS2185* (pBHR2) and JW270 *ΔBPSS2185* (pBHR2-*BPSS2185*) were used for the experiments described here. There was no growth difference in LB broth for any of the strains (data not shown). To identify the function of this type IVB locus, we looked at adhesion and biofilm formation capabilities.

**Table 2.** Transcriptomic data of *B. pseudomallei* genes upregulated in the presence of CaCl_2_ Genes represented are those with a *P*-value <0.05 and at least a 1.5 log fold change (FC).

Locus tag	NCBI ref. seq	LogFC	*P*-value	Protein function
*BPSS2186*	NC_006351.1	6.25132	0.006138	Pilus subunit
*BPSS2185*	NC_006351.1	5.58863	0.021329	Pilus subunit
*BPSS0092*	NC_006351.1	2.75258	0.028668	Fimbriae-related chaperone
*BPSL1627*	NC_006350.1	1.68243	0.03835	Fimbriae assembly chaperone
*BPSS0019*	NC_006351.1	1.60786	0.047893	Transport related membrane protein

### *BPSS2185* is required for adhesion to epithelial cells

**Fig. 2.**
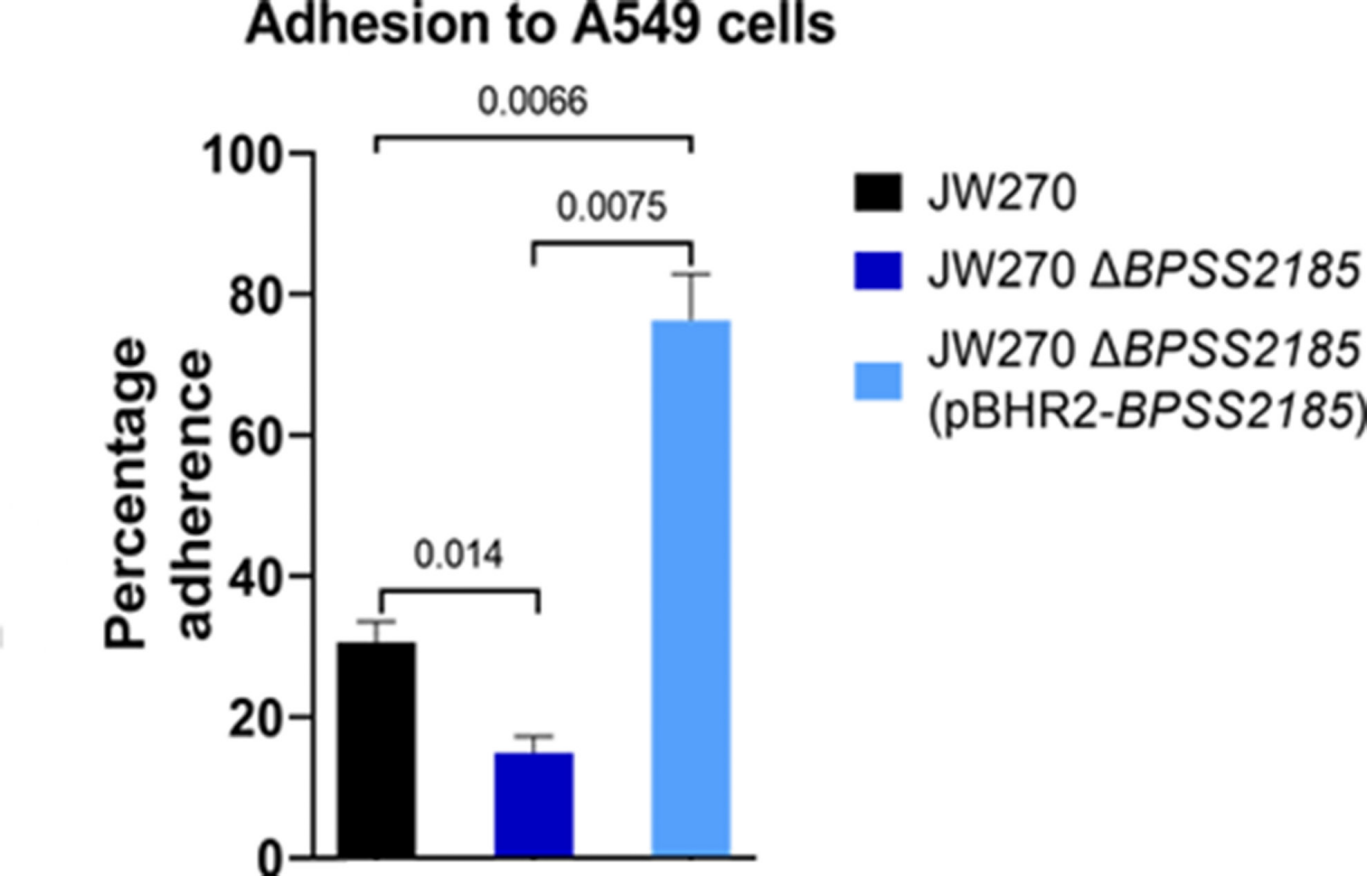
BPSS2185 facilitates adherence to A549 epithelial cells. Graph showing that the deletion of *BPSS2185* significantly reduced the ability to bind to A549 epithelial cells. Complementation of the *∆BPSS2185* mutation not only restored, but also enhanced, the adhesive capability of the mutant strain. Graph bars are representative of three independent experiments and error bars represent the standard error of means. A Student’s *t*-test was conducted to compare groups. *P*-values are indicated on the graph.

Adherence is required for cell or organ colonization [38]. To determine if BPSS2185 has an adherence function, we investigated the rate of adhesion to a biotic surface (A549 human epithelial cells) and an abiotic surface (96-well polystyrene plate). When compared to JW270, the ability of JW270 *ΔBPSS2185* to attach to A549 alveolar epithelial cells was reduced by about 50 % (Fig. 2, *P*=0.014). In contrast, JW270 *ΔBPSS2185* (pBHR2-*BPSS2185*) had a considerably higher adherence capability than both JW270 (*P*=0.0066) and JW270 *ΔBPSS2185* (*P*=0.0075). A control JW270 *ΔBPSS2185* (pBHR2) strain did not differ from JW270 *ΔBPSS2185,* ruling out influence from the empty vector in adhesion to A549 (data not shown). The increased copy number of the pilus subunit gene in the complemented mutant may have mediated the increased adherence phenotype. After 24 h on abiotic surfaces, the numbers of JW270, JW270 *ΔBPSS2185* and JW270 *ΔBPSS2185* (pBHR2-*BPSS2185*) cells adhering to 96-well plates were not significantly different (data not shown). While *BPSS2185* expression was higher when *B. pseudomallei* was grown in LB+1.26 mM CaCl_2_ as compared to LB (Table 2), we found that the expression of *BPSS2185* grown in LB media without CaCl_2_ was sufficient for assessing the phenotype of BPSS2185 in the adherence assay and all subsequent experiments described below.

### Twitching motility assay

**Fig. 3.**
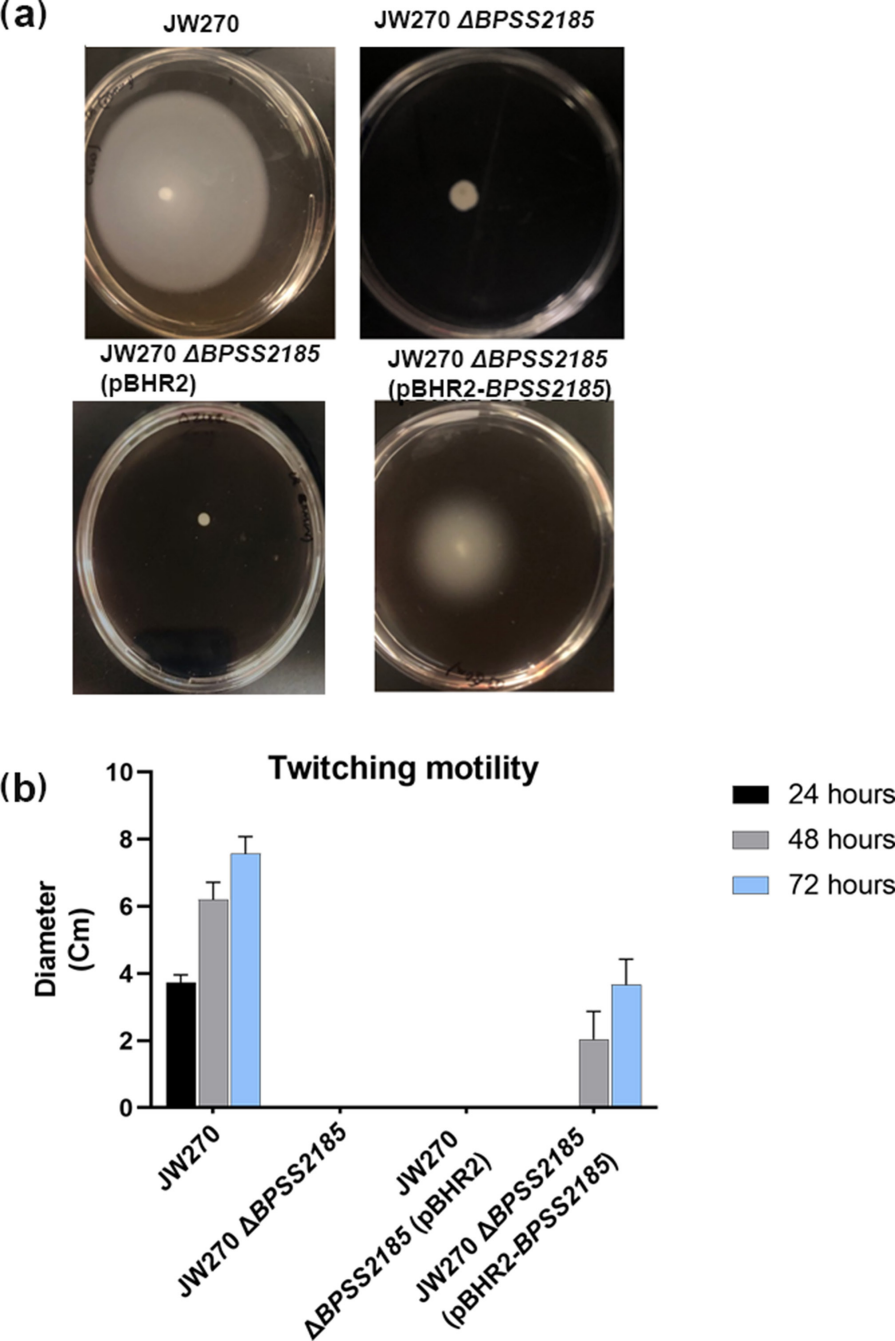
BPSS2185 is required for *B. pseudomallei* twitching motility. (**a**) Agar plate images showing twitching motility of the strains at 48 h. No halo was observed for JW270 *ΔBPSS2185*. The twitching motility phenotype of JW270 *∆BPSS2185* was partially complemented by pBHR2-*BPSS2185*, but not pBHR2. (**b**) Graph showing distance twitched by each strain represented in Fig. 3(a). Twitching motility is measured as the diameter of the halo (cm). Bars are an average of three independent experiments. Error bars are the standard error of means.

Fig. 3 shows the ability of the bacteria to twitch from an initial point of inoculation and form a circular white halo after 3 days of growth. The diameter of the halo was measured to assess the relative twitching motility of each bacterial strain examined. *B. pseudomallei* JW270 is a motile strain that formed a twitching halo with a diameter of 4 cm by day 1, 6 cm by day 2 and 8 cm by day 3, but JW270 *ΔBPSS2185* does not move from the point of inoculation (Figs. 2 and 3). This loss of function was partially restored in JW270 *ΔBPSS2185* (pBHR2-*BPSS2185*), which formed a twitching halo with a diameter of 2 cm by day 2 and 4 cm by day 3.

### BPSS2185 influences biofilm formation

**Fig. 4.**
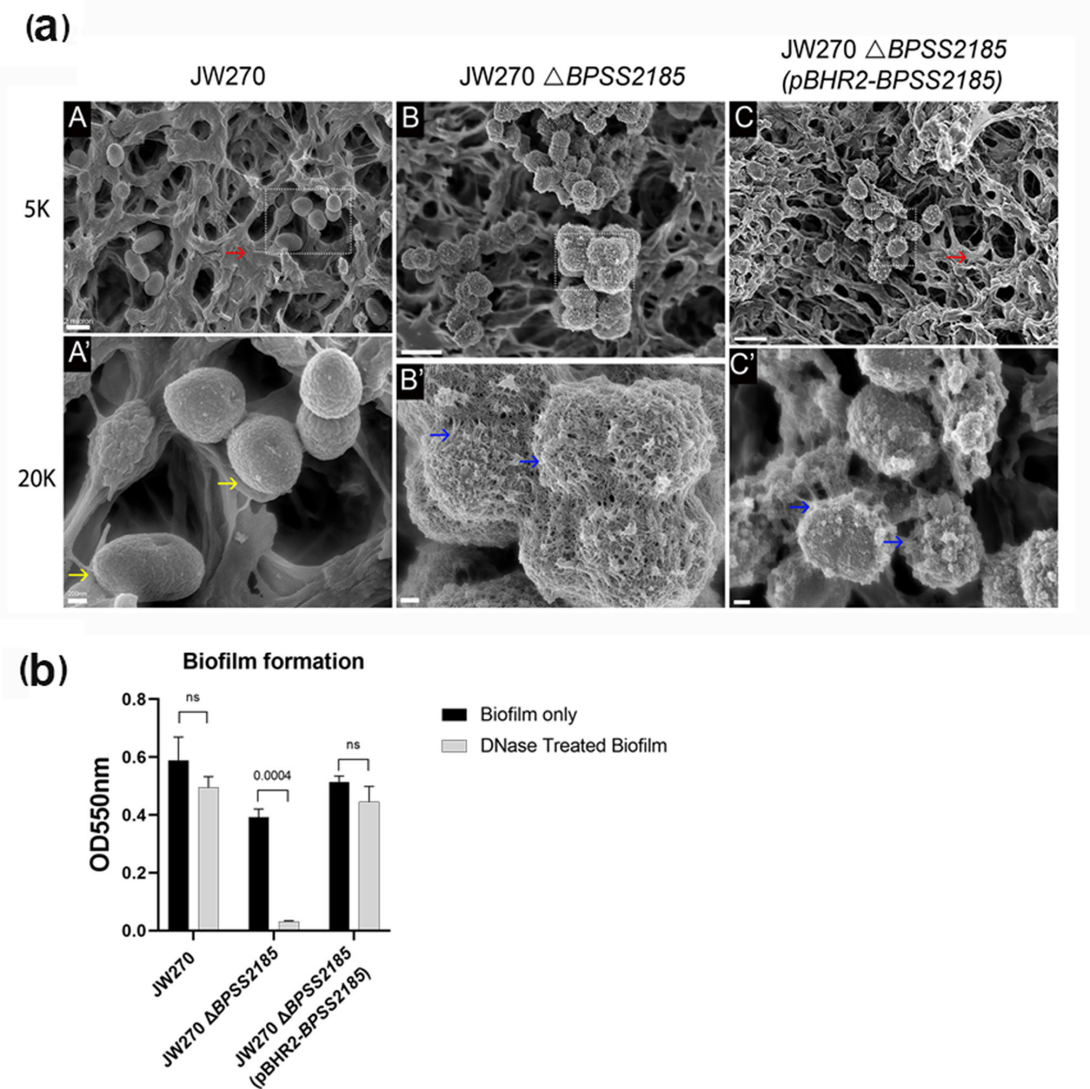
Biofilm formation by *B. pseudomallei *. (**a**) Scanning electron microscope imaging of biofilms formed by *B. pseudomallei* strains on day 3. The same image for each strain is shown at 5000× (top row) and 20 000× (bottom row). A scale bar for the entire row is shown at the left of each row. Top row: red arrows indicate a robust biofilm. Bottom row: yellow arrows indicate a bare bacterial cell and blue arrows show non-confluent biofilm. (**b**) Biofilm formed by all three strains on day 3 before and after treatment with DNAse. Each bar is an average of three independent experiments and the standard error of means are represented as error bars. All groups were compared using *t*-tests.

SEM images from a 3 day biofilm grown on nitrocellulose membrane are presented in Fig. 4(a). The top and bottom row images are the same images at different magnifications. In the top row, both JW270 and JW270 *ΔBPSS2185* (pBHR2-*BPSS2185*) were able to form a solid mass of biofilm (red arrows) which covers individual bacterial cells (yellow arrow, bottom row). We observed that the bacterial cells in JW270 *ΔBPSS2185* and some JW270 *ΔBPSS2185* (pBHR2-*BPSS2185*) cells were visually coated by an adhesive structure or a weak biofilm (blue arrow, bottom row). A biofilm assay using CV did not show any significant differences in biofilm formation (*P*=0.586, Fig. S1, available in the online version of this article), but the biofilm formed by JW270 *ΔBPSS2185* significantly responded to treatment with DNase (Fig. 4b, *P*=0.004, JW270 *ΔBPSS2185* pre- and post-DNase treatment), indicating that the biofilm observed in Fig. 4(a) by this strain may be mostly eDNA in composition.

### eDNA export increases in JW270 *ΔBPSS2185*

**Fig. 5.**
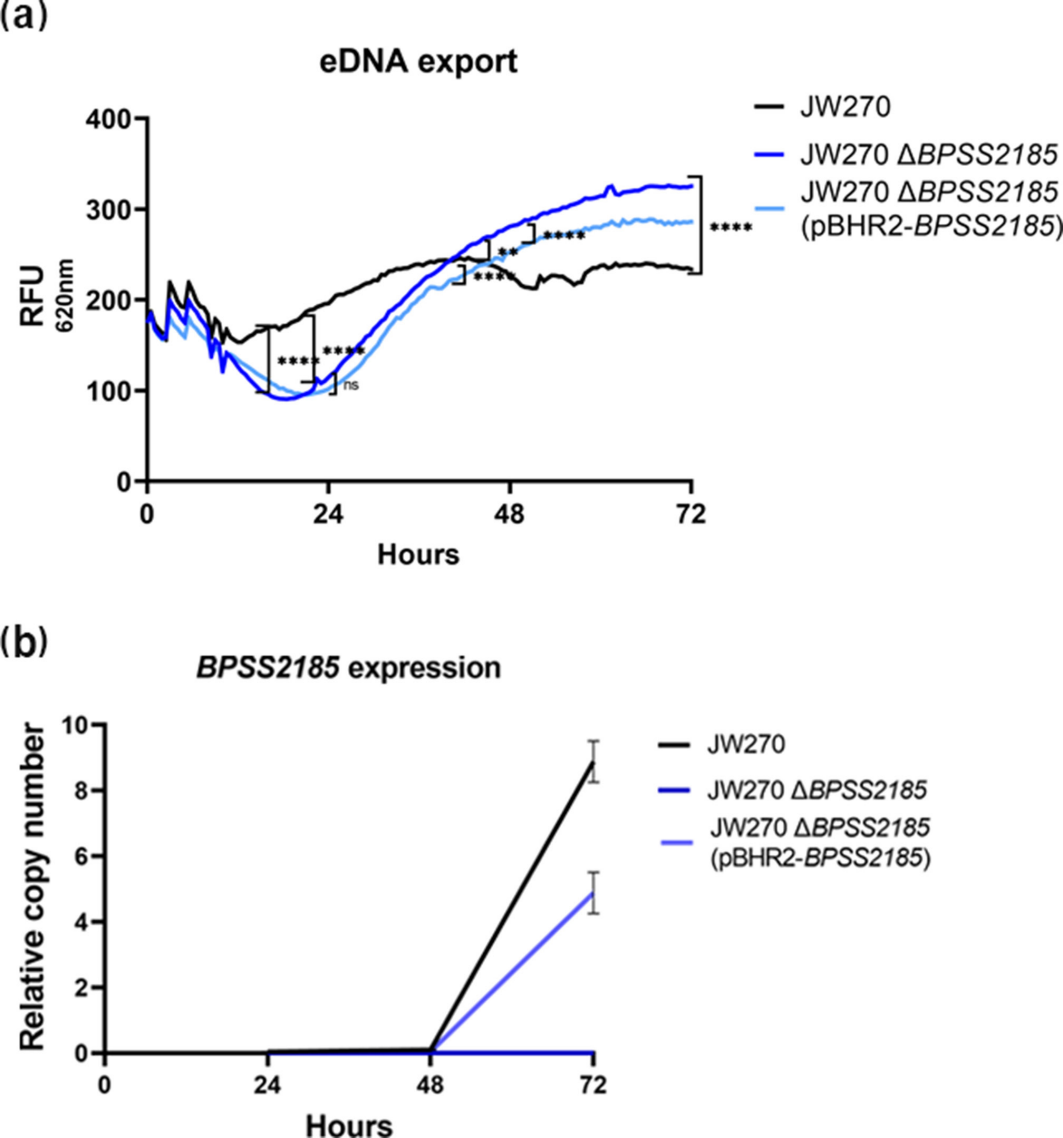
eDNA export and *BPSS2185* expression in JW270. (**a**) A 72 h representation of eDNA export by JW270, JW270 *ΔBPSS2185* and JW270 *ΔBPSS2185* (pBHR2-*BPSS2185*) biofilms. Data are the mean of three replicates per strain and readings were taken at 30 min intervals for 72 h. A one-way ANOVA was used to analyse all three groups. The *P* values for the 24, 48 and 72 h time points are indicated by brackets and asterisks for all strains on the graph.**P=0.0026; ***P≤0.0001; ns, not significant. At 72 h, all strains were significantly different from one another (*P*=0.0001). (**b**) RT-qPCR showing expression of JW270 *BPSS2185*, JW270 *ΔBPSS2185* and JW270 *ΔBPSS2185* (pBHR2-*BPSS2185*).

eDNA is an essential component of *B. pseudomallei* biofilm [39]. The eDNA export by JW270, JW270 *ΔBPSS2185* and JW270 *ΔBPSS2185* (pBHR2-*BPSS2185*) was continuously quantified at 30 min intervals over a 72 h period (Fig. 5a). JW270 exported significantly more eDNA than the other strains by 24 h, but at 72 h both JW270 *ΔBPSS2185* and JW270 *ΔBPSS2185* (pBHR2-*BPSS2185*) exported significantly more eDNA than JW270 (*P*=0.0001). JW270 *ΔBPSS2185* (pBHR2-*BPSS2185*) exported significantly less eDNA than JW270 *ΔBPSS2185* at 72 h (*P*=0.0001)*,* which suggests that BPSS2185 has a negative influence on eDNA export (Fig. 5a). We confirmed that PI fluorescence was from viable cells; a 72 h live/dead microscopic image indicated that all cells were mostly viable, signifying that the increase in eDNA/PI fluorescence was active and not the result of bacterial mortality (Fig. S2).

### *BPSS2185* expression

The JW270 *ΔBPSS2185* biofilm scanning electron micrographs show weakened biofilms (Fig. 4a) that were sensitive to DNase treatment (Fig. 4b), and that JW270 *ΔBPSS2185* exported more eDNA than the parental and complementation strain (Fig. 5a). We studied the expression of *BPSS2185* during eDNA export. On days 1 and 2, *BPSS2185* was not expressed in any of the strains. Day 3 of the eDNA export coincides with the formation of a mature biofilm in JW270 and JW270 *∆BPSS2185* (pBHR2-*BPSS2185*) (Fig. 4a), and *BPSS2185* expression increased four- and nine-fold for JW270 and JW270 *ΔBPSS2185* (pBHR2-*BPSS2185*), respectively (Fig. 5b). JW270 *ΔBPSS2185* served as a negative control as the gene was deleted in-frame and no expression was expected.

## Discussion

Bacterial adhesion and biofilm formation are implicated as major factors for disease progression and persistence [14]. Bacterial adhesion is the first interaction of a microbe and host cell and ultimately leads to biofilm formation and infection. As a result, adhesion interference/anti-adhesion therapy has been suggested as an effective means of preventing or treating bacterial infections [14, 40, 41]. The biofilm-forming strains of *B. pseudomallei* are highly virulent [26] and *B. pseudomallei* biofilms facilitate adhesion and intracellular survival in epithelial cells [42]. Adhesion of *B. pseudomallei* to airway epithelial cells followed by internalization is vital for pathogenesis and bacteria–host interactions [42].

We applied genetic deletion and complementation analysis to this *B. pseudomallei* JW270 gene to characterize its contribution to biotic and abiotic adherence, twitching motility, and biofilm formation. Using RNA-Seq and transcriptomic analysis, we identified the *BPSS2185* TFP8 gene that encodes a type IVB pilin subunit protein. Out results suggest that the TFP8 locus, specifically *BPSS2185*, plays a role in these phenotypes, which may influence *B. pseudomallei* virulence and persistence.

The TFP8 locus, *BPSS2185–BPSS2198*, encodes two pilin subunit proteins, BPSS2185 and BPSS2186 (Fig. 1). In order to study the TFP8 pilus subunit genes, we attempted to construct mutations in both *BPSS2185* and *BPSS2186*. Deletion of *BPSS2186* proved unsuccessful after three attempts, but we were able to generate *B. pseudomallei* JW270 *ΔBPSS2185* and JW270 *ΔBPSS2185* (pBHR2-*BPSS2185*), a complementation strain. *BPSS2185* is a part of the TFP8 locus in *B. pseudomallei* and this locus has been suggested as a recombination hotspot and implicated in modulating virulence in *B. pseudomallei* [13]. Nandi *et al*. showed that a TFP8 deletion mutant exhibited significantly reduced virulence in a murine intranasal infection assay compared to a parental *B. pseudomallei* K96243 wild-type control [13]. Likewise, our data suggest that *BPSS2185* is required for full epithelial cell adhesion (Fig. 2) and twitching motility (Fig. 3), two characteristics required for full virulence and the formation of a robust biofilm (Fig. 4). *BPSS2185* is suppressed during biofilm formation (Fig. 5b), but increased in a mature biofilm to aid with new cell colonization, as noted in the literature with other motile genes [28–30].

Adhesion of *B. pseudomallei* to airway epithelial cells followed by internalization is vital for pathogenesis and bacteria–host interactions [42]. *B. pseudomallei* biofilms facilitate adhesion and intracellular survival in epithelial cells, leading to chronic and difficult to eradicate infections [42]. When JW270 *ΔBPSS2185* was grown in the presence of A549 epithelial cells, we observed that the deletion mutant failed to adhere efficiently to the cells. There was about a 50 % reduction in the percentage of cells that adhered in comparison to the parent JW270. The complementation strain, *B. pseudomallei* JW270 *ΔBPSS2185* (pBHR2-*BPSS2185*), presented increased A549 adhesion. This study showed no significant difference in adhesive properties on abiotic surfaces for JW270 and JW270 *ΔBPSS2185,* suggesting that BPSS2185 is involved with adhesion to cells, but not abiotic surfaces. Therefore, the inability of the JW270 *ΔBPSS2185* strain to effectively adhere or form a biofilm may be the mechanism of attenuated virulence demonstrated by Nandi *et al.* [13].

Twitching motility is a ﬂagella-independent form of bacterial movement over surfaces which occurs through pili extension and retraction and involves a type IV pilus [43, 44]. Twitching motility is also a means of rapid colonization of new surfaces by bacterial communities [45]. Genes implicated in twitching capabilities are required for chronic infection and biofilm formation in Gram-negative bacteria [28, 46]. Chronic infections occur when planktonic bacteria are dispersed from a biofilm, and twitching genes are required for the planktonic bacteria to move to a new location and form a new biofilm [47]. In Fig. 3(a, b), we show that deletion of *ΔBPSS2185* attenuated the ability of the bacteria to twitch, a function that was partially restored once the gene was complemented in JW270 *ΔBPSS2185* (pBHR2-*BPSS2185*). Recently, an oligosaccharyltransferase, PglL, was linked to twitching motility in *B. pseudomallei* [48], but our study is the first to actively implicate BPSS2185 in twitching motility.

BPSS2185 shares 43 % similarity with Flp-1 in *Aggregatibacter actinomycetemcomitans* [9]. In *A. actinomycetemcomitans *, Flp-1 is required for non-specific adherence [49] and an *flp-1* mutant exhibited reduced surface adherence much like our *B. pseudomallei* JW270 *∆BPSS2185* data shown in Fig. 2. The *BPSS2185*-encoded pilin is closely related to Flp-1 of *A. actinomycetemcomitans* and it is involved with forming a strong biofilm, as indicated by *B. pseudomallei* JW270 and JW270 *ΔBPSS2185* (pBHR2-*BPSS2185*) [50]. Disruption of *A. actinomycetemcomitans flp-1* resulted in a mutant that formed a weakly adherent biofilm [49], similar to what was observed with JW270 *ΔBPSS2185* (Fig. 4a). Our biofilm results suggest that the *ΔBPSS2185* mutation was only partially complemented by pBHR2-*BPSS2185*, which supports and is consistent with our finding that complementation partially restored twitching motility.

*B. pseudomallei* biofilms are made up of proteins, polysaccharides and eDNA, and we assessed eDNA export in strains with and without *BPSS2185* over a period of 72 h (Fig. 5a). Surprisingly, JW270 *ΔBPSS2185* exported more eDNA than JW270 at 48 and 72 h. We also observed that the amount of eDNA exported by JW270 *ΔBPSS2185* (pBHR2-*BPSS2185*) was decreased relative to JW270 *ΔBPSS2185*, suggesting that BPSS218*5* may impact eDNA export negatively. The mechanism by which eDNA is exported by *B. pseudomallei* is currently unknown and the inhibition of eDNA export, either directly or indirectly, by type IV pili has not been described in other bacterial species. We speculate that while eDNA export is important during the early stages of biofilm formation, twitching motility is less important and *BPSS2185* expression is low. As *B. pseudomallei* cells are dispersed during biofilm expansion, BPSS2185-mediated twitching motility is required and *BPSS2185* expression increases while eDNA export becomes less important and is dampened down. Further studies will be necessary to understand these processes at the molecular level.

In summary, our study is the first to demonstrate a potential mechanism by which TFP8 contributes to *B. pseudomallei* virulence. By applying genetic deletion and complementation analysis to the *B. pseudomallei BPSS2185* pilin gene, we have shown that the gene is necessary for the bacteria to twitch, colonize epithelial cells and form a tenacious biofilm. We propose that TFP8 participates in the dispersal of *B. pseudomallei* from mature biofilms and contributes to the formation of new biofilms, but future experiments will be necessary to definitively prove this.

### Data availability

The authors declare that the data supporting the findings of this study are available within the paper (and its supplementary files). The RNA-Seq data have been deposited in Figshare (accession number: https://figshare.com/articles/dataset/Burkholderia_pseudomaelli/17704376).

## Supplementary Data

Supplementary material 1
