# Supplementary material for: A type IVB pilin influences twitching motility and in vitro adhesion to epithelial cells in Burkholderia pseudomallei
Source: Microbiology (Reading). 2022 Mar 16;168(3):001150. doi: 10.1099/mic.0.001150 (PMC9558350; doi:10.1099/mic.0.001150)
Supplement: Supplementary material 1 [file mic-168-1150-s001.pdf]

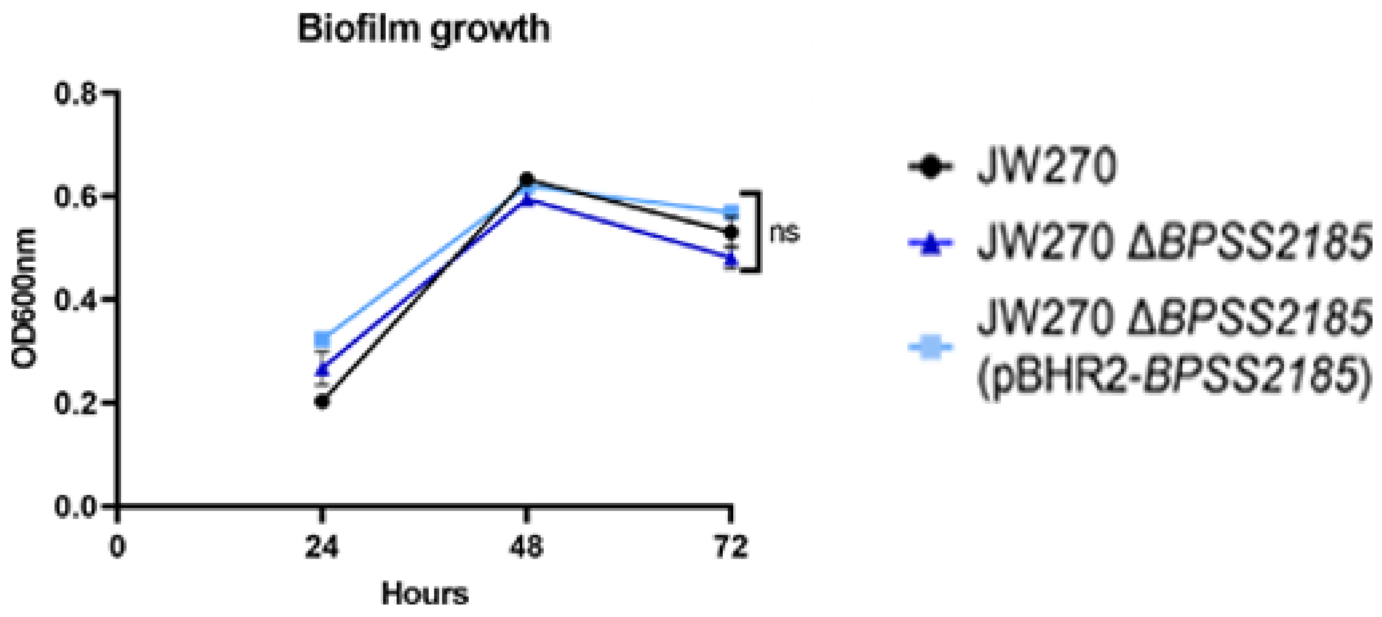

Figure S1. Biofilm formation for three *Burkholderia pseudomallei* strains. There was no significant difference in biofilm formed. ns, not significant.

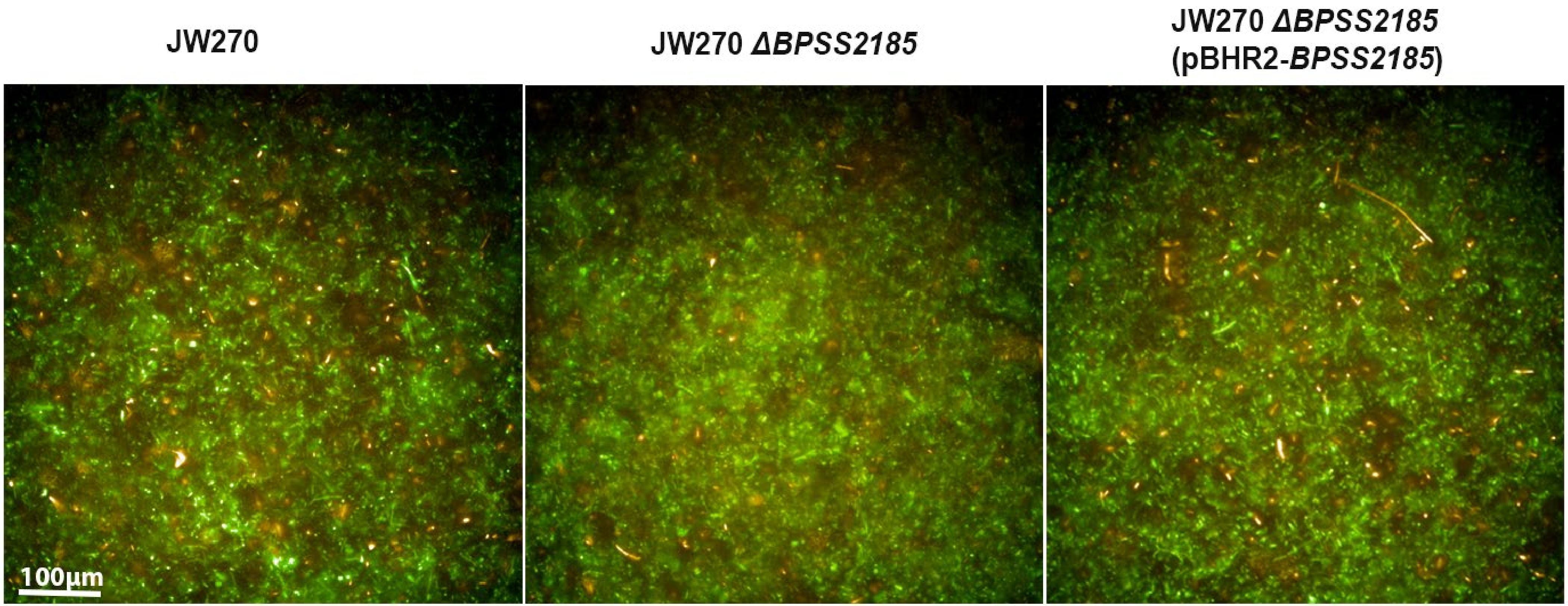

Figure S2. Fluorescent microscope images for the cells washed and stained with SYBR green to determine viability of cells after eDNA export in Fig. 5a. A scale bar of 100 µm is shown in white at the bottom left.
